# An unusual presentation of urethral duplication presenting with chronic bladder retention, left scrotal transposition and left renal agenesis

**DOI:** 10.1590/S1677-5538.IBJU.2016.0119

**Published:** 2018

**Authors:** Antonio Macedo, Marcela Leal da Cruz, João Luiz Gomes Parizi, Gustavo Marconi Caetano Martins, Riberto Liguori, Sérgio Leite Ottoni, Bruno Leslie, Gilmar Garrone

**Affiliations:** 1 Universidade Federal de São Paulo, São Paulo, Brasil

## Abstract

**Introduction and objective:**

Urethral duplication is a rare congenital anomaly, with roughly 200 cases reported in the literature (1). It is more frequent in males, with few cases reported in females. The clinical presentation differs according to the anatomical variant present. The duplication most commonly occurs in the sagittal plane with one urethra located ventrally and the other dorsally (2). Usually the ventral urethra is the more functional of both. Duplications occurring in the coronal plane are quite rare and they are usually associated with bladder duplication (3). The purpose of this paper was to present a video of a boy with an unusual urethral duplication form.

**Materials and Methods:**

Patient was born premature due to oligohydramnios at 7 months-gestational age and he has initial diagnosis of hypospadia. Since then, he presented at least 7 febrile UTI and mother complained of difficult micturition and a presence of a mass at lower abdomen. Patient was referred to our institution and we identified urethral duplication with a glandar and scrotal meatus, palpable bladder and left penile-hemiscrotum transposition. US and CT-scan showed left kidney agenesis and overdistended bladder. VCUG and retrograde urethrography showed duplication, presence of contrast in the seminal vesicles and complete catheterizing of both urethras was not possible.

**Results:**

The topic urethra was dysplastic and not patent to a 4Fr plastic tube so we were unable to access it endoscopically. We performed initially a Mitrofanoff procedure to allow CIC and treat chronic retention. Six months later, we assessed both urethras surgically and concluded that dorsal urethra was dysplastic after 3cm still in the penile area and scrotal urethra was not possible to be catheterized. We excised the ventral urethra because of dribbling complaints up to bulbar area and reconstructed the scrotal transposition, keeping the topic urethra for cosmetic issues. Patient had excellent outcome, performs CIC every 4 hours and has not presented further UTI episodes.

**Discussion and conclusion:**

The urethral duplication is an anomaly that has multiple anatomical presentations. There are several theories about the etiology, but none can explain all types of presentations. There is also more than one rating available, and the Effmann classification is the most detailed. The case exemplifies this varied spectrum of anatomic urethral duplication. It resembles the urethral duplication type IIa-Y, however, ventral urethra meatus was located in penoscrotal area and both urethras were at least partially hypoplastic/dysplastic associated with obstruction and bladder retention. In determining how to best manage a patient with Y-type urethral duplication, the caliber and quality of the orthotopic urethra must first be assessed. Published reports suggest that best outcomes are those using the ventral duplicated urethra for the reconstruction (4). In this case, none of urethras were functional and a supravesical outlet channel had to be provided. The treatment of this condition requires an individualized planning and a vast technical knowledge of reconstructive surgery.

## ARTICLE INFO

**Available at: http://www.intbrazjurol.com.br/video-section/20160119_Macedo_et_al**

**Int Braz J Urol. 2017; 44 (Video #7): 409-10**
